# Fructose Promotes Crucian Carp Survival Against *Aeromonas hydrophila* Infection

**DOI:** 10.3389/fimmu.2022.865560

**Published:** 2022-03-21

**Authors:** Yunchao Cao, Tianshun Kou, Liaotian Peng, Hetron Mweemba Munang’andu, Bo Peng

**Affiliations:** ^1^ State Key Laboratory of Biocontrol, Guangdong Key Laboratory of Pharmaceutical Functional Genes, School of Life Sciences, Southern Marine Science and Engineering Guangdong Laboratory (Zhuhai), Sun Yat-sen University, Guangzhou, China; ^2^ Laboratory for Marine Biology and Biotechnology, Qingdao National Laboratory for Marine Science and Technology, Qingdao, China; ^3^ Department of Biosciences and Aquaculture, Nord University, Bodø, Norway

**Keywords:** fructose, crucian carp, *Aeromonas hydrophila*, metabolomics, bacteria clearance

## Abstract

Aquatic food is becoming an important food source that provides micronutrients to human beings. The decline of wild aquatic animals makes aquaculture become increasingly important to play this role. However, infectious diseases, especially bacterial infection, represent severe threat to aquaculture, which causes huge economic loss. Meanwhile, strategies in managing bacterial infection in an antibiotic-independent way are still lacking. In this study, we monitor the metabolomic shift of crucian carp upon *Aeromonas hydrophila* infection. We find that the metabolism of the fish that died of infection is distinct from the ones that survived. By multivariate analysis, we identify fructose as a crucial biomarker whose abundance is significantly different from the dying and surviving groups where the surviving group has a higher content of fructose than the dying group. Exogenous supplementation of fructose increases fish survival rate by 27.2%. Quantitative gene expression analysis demonstrated that fructose enhances the expression of lysozyme and complement 3 expression, which is also confirmed in the serum level. Furthermore, the augmented lysozyme and C3 levels enhance serum cell lytic activity which contribute to the reduced bacterial load *in vivo*. Thus, our study demonstrates a metabolism-based approach to manage bacterial infection through modulating immune response to clear bacterial infection.

## Introduction

Bacterial infectious diseases are still one of the major factors that threaten human health and pose big challenges to sustainable poultry industries worldwide. Aquaculture industries are expected to meet the increasing demand for high-quality protein in the future (1, 2). However, around 6 million dollars are lost in aquaculture annually due to infectious diseases, and bacterial infections account for half of the deaths of aquatic animals (3, 4). *Aeromonas* spp. represent species that are highly pathogenic to aquatic animals and can be isolated globally in diseased aquatic animals (5, 6). *Aeromonas hydrophila*, for example, is an important, motile, rod-shaped opportunistic bacteria, which is found in waters all over the world and has been detected in various meat, vegetables, and seafood, posing potential threat to food safety. *A. hydrophila* infect various types of fish, including common carp, gold fish (7), yellow catfish (8), and channel catfish (9). Moreover, the main symptoms of the fish being infected by *A. hydrophila* is associated with necrosis, ascitic fluid, and darkening of the spleen and kidney and pale liver (10), and the fish dies within 3 days (11). *A. hydrophila* is also pathogenic to human beings by causing gastroenteritis that exhibits abdominal pain, nausea, vomiting, and diarrhea (12, 13).

Broad-spectrum antibiotics including tetracyclines/cefotaxime and quinolones were routinely used to treat *A. hydrophila* infection (14). However, the misuse and overuse of antibiotic increase the prevalence of antibiotic resistance and the global spread of antibiotic-resistant genes, limiting the therapeutic effects of antibiotics (15). Antibiotic-resistant *A. hydrophila* are frequently isolated from environments and aquaculture ponds. Several recent studies mapped the resistant profiles of *A. hydrophila* isolated from aquatic animals. The resistant rates of these strains were from 6.98% to 100% for quinolones, from 23.36% to 92.31% for tetracyclines, and from 61.54% to 100% to β-lactams (16). Therefore, antibiotics should be used with caution since antibiotics can easily contaminate the environment *via* water circulation (17). Seeking for alternative approaches is urgently required.

Harnessing the host’s immune system has been an attractive strategy to prevent or treat bacterial infection (18). Although vaccines have been proved to be one of the most successful approaches in preventing infectious diseases, the progress is still limited in aquaculture. In China, for example, there are only 6 certified vaccines to be used in aquaculture (19), which dramatically lags the demand to treat various types of pathogens. We recently proposed that reprogramming metabolomics is a promising approach to modulate host immune response to bacterial infection (20–22). This strategy is based on the idea that the metabolic states of the fish determine their consequences of infection, which can be attributed to the strong relationship between metabolism and immunity, whereas the reprogramming of the metabolome of the fish may drive their immune response to fight against bacterial infection (23). This concept has been well studied in tilapia and zebrafish (24). Glucose increased fish survival by 14% to 60% upon *Edwardsiella tarda* infection (25, 26). L-Aspartic acid, L-leucine, and L-valine increase tilapia survival against both gram-positive and -negative pathogens (27–30). Moreover, other metabolites such as myo-inositol (31), maltose (32), l-proline (29), and malate (33) increase important aquatic fish—such as crucian carp—survival rates by 14% to 60%. This approach was also used for reversing antibiotic resistance (21, 34–38). Meanwhile, the exploration of cell-mediated immune and antibody-dependent clearance of bacterial pathogens also sheds light on novel targets to treat bacterial infections (39–42). For example, IgT emerged as a key factor in defending against bacterial pathogens, which are present in the mucosa of teleost fish (43).

Crucian carp, *Carassius carassius*, is one of the most economically important freshwater fishes in the world. The production reached 2.2 million tons in 2010 according to FAO statistical data (44). Meanwhile, crucian carp is a good model for the assessment of aquatic ecosystems, for various toxicological studies (45), and more importantly, as infection host to study host–pathogen interactions (46).

In this study, we investigate the metabolic response of *C. carassius* to *A. hydrophila* infection at LD_50_. The surviving fish and dead fish showed differential metabolic responses, and this difference contributes to the immune response. Therefore, we identified fructose as a crucial biomarker, whose abundance was decreased in the dying fish. Administration of fructose to the fish increased their survival from 46.67% to 76.67%. Moreover, the mechanism involves increased lysozyme and C3 activity. Data are shown as follows.

## Material and Methods

### Ethics Statement

Animal studies involved in this manuscript adhere to the recommendations in the Guide for the Care and Use of Laboratory Animals of the National Institutes of Health and maintained according to the standard protocols. All the experiments were reviewed and approved by the Institutional Animal Care and Use Committee of Sun Yat-sen University (Approval No. SYSU-IACUC-2020-B126716).

### Bacterial Culture and Crucian Carp Maintenance

*Aeromonas hydrophila* LP-2 was a generous gift by Prof. X. M. Lin from Fujian Agriculture and Forestry University. The bacteria were confirmed by 16S rDNA sequencing. The bacteria were stored at -80°C and cultured in Luria-Bertani medium at 30°C. Crucian carp with average weight 3 g ± 0.5 g were obtained from a local crucian carp-breeding corporation (Panyu District, Guangzhou, China) and were free of *Aeromonas* infection through microbiological detection. The fish were maintained in a 170-l water tank equipped with a closed recirculating aquaculture system and fed once a day with commercial fish food. The fish culture room was equipped with normal light at a rhythm of 12-h/12-h light and darkness.

### Bacterial Infection

For bacterial infection, a single colony of *A. hydrophila* was picked up from the agar plate and grown in LB medium overnight at 30°C shaken at 200 rpm. The overnight culture was then reinoculated to fresh LB medium at a ratio of 1:100 and grown with shaking at 200 rpm until OD600 reached 1.0. Cells were pelleted by centrifugation, washed with 0.85% sterilized saline solution for three times, and resuspended in the same solution.

For bacterial infection, *Carassius carassius* were individually challenged with 5 μl of 1 × 10^6^ CFU bacterial suspension or saline only *via* intraperitoneal injection (n = 20 for each treatment). Fish mortality was examined twice daily for 10 days to monitor accumulative death. The dying crucian carp were selected when they had the following symptoms: darkening body, slow and unbalanced swimming, and swimming close to the water surface; the crucian carp that survived after 3 days, were swimming smoothly, and had a normal body color were defined as survived fish.

### Sample Preparation for Gas Chromatography–Mass Spectrometry Analysis

The sample preparation was carried out as previously described (32, 47). Individual livers from each fish were surgically removed and quickly rinsed with cold saline buffer to remove blood. Then, cold methanol was added at 800 μl/100 mg. The whole liver was homogenized in methanol, followed by sonication for 5 min at a 10-W power setting. A total of 30 C*. carassius* accommodated at 26°C were used in this experiment (n = 10 for each group). The liver from individual *C. carassius* was treated as one biological sample. Samples were then centrifuged to remove unresolved matter at 12,000 × g, 4°C for 10 min. The supernatant was collected, and 10 μl 0.1 mg/ml ribitol (Sigma-Aldrich, St. Louis, MO, USA) was added as an internal standard. Afterward, the aqueous sample was concentrated in a rotary vacuum centrifuge device (Labconco, Kansas City, MO, USA) for 4 h, and the resultant dried extracts were used for gas chromatography–mass spectrometry (GC-MS) analysis.

### GC-MS Analysis

The GC-MS analysis was carried out following the two-stage techniques with minor modifications (10). In brief, samples were derivatized in 80 μl of 20 mg/ml methoxamine hydrochloride (Sigma-Aldrich, USA) in pyridine to protect carbonyl moieties through methoximation for 90 min at 37°C, followed by addition of 80 μl of N-methyl-N-trimethylsilyl-trifluoroacetamide (MSTFA) (Sigma-Aldrich, USA) for derivatization of acidic protons at 37°C for another 30 min. The derivatized sample of 1 μl was injected into a 30 m × 250 μm i.d. × 0.25 μm DBSMS column using splitless injection, and analysis was carried out in Agilent 7890A GC equipped with an Agilent 5975 C VL MSD detector from Agilent Technologies (Santa Clara, CA, USA). The initial temperature of the GC oven was held at 85°C for 5 min followed by an increase to 270°C at a rate of 15°C min^−1^, and then held for 5 min. Helium was used as the carrier gas, and the flow rate was kept constantly at 1 ml min^−1^. MS was operated in a range of 50–600 m/z. For each sample, two technical replicates were prepared to confirm the reproducibility of the reported procedures.

### Exogenous Addition of Fructose and Bacterial Challenge

*C. carassius* (n = 90) were randomly divided into three groups, acclimatized for 7 days at 28°C. Thirty individuals were included in each group. As the fish were of similar weight, each fish was injected with 10 μl 0.85% sterilized saline solution as control group or with 50 and 100 μg fructose as the experimental groups. The fish were injected once daily for 3 days. After the treatment, fish were challenged by intraperitoneal injection of 6.25 × 10^6^ CFU/fish *A. hydrophila*. Crucian carp were observed for 10 days for accumulative death.

### RNA Isolation and qRT-PCR

Total RNA of liver was isolated with TRIzol (Invitrogen, Carlsbad, CA, USA). The isolated RNA was then quantified by detecting the intensity of fluorescence. Reverse transcription-PCR was carried out on a Primer-Script™ RT reagent kit with a gDNA eraser (Takara, Japan) with 1 μg of total RNA according to the manufacturer’s instructions. The experiment was performed on six or ten biological samples obtained. qRT-PCR was performed as described previously (47). Primers for each gene are listed in **Supplementary Table 1**. Each primer pair was specific. The relative expression of each gene was determined by the comparative threshold cycle method (2^−ΔΔCT^ method) (48).

### Serum Lysozyme, Complement, and Bactericidal Activity

After treatment with fructose or sterilized saline solution, fish (n = 10 for each group) were anaesthetized on ice for 10 min to slow the movement of the fish. Blood samples were collected with sterile syringes from the caudal vein around the caudal peduncle into 1.7-ml microcentrifuge tubes without anticoagulants. Collected samples were kept at room temperature for 30 min to allow blood clotting and kept at 4°C overnight. On the second day, blood samples were centrifuged and the supernatant was pipetted into a sterile 1.5-ml microtube. The samples were aliquot and stored at -80°C until use.

For serum lysozyme activity, measurement was performed as was determined by turbidimetric assays as described by Caruso et al. (49) and followed the guidance of Lysozyme Activity Kit (Jiancheng, China). 200 μl of standard lysozyme or serum sample was pipetted into a 5-ml sterilized centrifuge tube and mixed with 2 ml diluted *Micrococcus lysodeikticus* suspension for less than 10 s to complete the system and measured OD530 in a well-cleaned cuvette. Observation values were taken as T0 and T10 when the cuvette was measured instantly and 10 min later. The lysozyme activity was calculated as the following:


Lysozyme activity (U/mL)=Sample (T10−T0)/standard (T10−T0)∗standard (U/mL)∗dilution fold.

For serum complement activity, alternative complement serum activity was determined as previously described (50, 51). Sheep red blood cells (ShRBC) were washed three times with sterilized pre-cold saline solution and resuspended with ethylene glycol tetraacetic acid–magnesium–gelatin veronal buffer (0.01 M EGTA–Mg–GVB, pH 7). ShRBC suspension was adjusted to 1 × 10^9^ cells/ml. 2 units of ShRBC antibody (Yuanye, China) were used to sensitize prepared ShRBC in a water bath at 37°C with shaking once per 5 min for 30 min. Sensitized ShRBC was washed with EGTA–Mg–GVB buffer two times and resuspended in the same volume of buffer. The complement lysis system including 1.5 ml diluted serum and 1 ml sensitized ShRBC was mixed gently and bathed at 37°C for 30 min. The CH50 standard sample was measured at 0.5 ml 2% ShRBC mixed with 2.5 ml distilled water and incubated at 37°C for 30 min. Then, the sample was centrifuged and the supernatant was collected and measured at OD542. The complement activity was as follows: CH50 (U/mL) = (1/serum volume/mL) * dilution fold.

For bactericidal effect, log-phase *A. hydrophila* bacterial cultures (OD600 = 0.5) were collected, washed with saline, and resuspended to OD600 = 0.2. 2 ml of the resuspended culture was centrifuged and resuspended in 200 μl serum isolated from either fructose-treated fish or saline-treated fish. The mixture was incubated at 30°C with shaking for 2 h. At last, 1.8 ml saline was added to the system to stop the reaction, which was subsequently used for plating.

### Liver Bacteria CFU Counting

To quantify the bacterial loads in the liver, the liver was isolated and collected in a 1.5-ml Eppendorf tube and was kept on ice. The tissue was thoroughly homogenized in 200 μl pre-cold saline buffer (10 mg/ml) by a grinding rod. Then, 100 μl homogenates was serially diluted for plating.

### Liver Mixture Bactericidal Effects

To assess the bactericidal effect of liver homogenates to *A. hydrophila*, livers from either saline or fructose-treated crucian carp were isolated and homogenized in pre-cold PBS buffer at 5% (m/v). Homogenates were centrifuged at 4°C and 5,000 rpm for 10 min, and the supernatant was collected. 200 μl *A. hydrophila* (OD600 = 0.2) bacterial culture was mixed with the supernatants at 30°C with shaking. At last, 1.8 ml saline was added to the reaction, which was used for plating by serial dilution.

## Results

### Crucian Carp Mount Differential Metabolic Response Upon *A. hydrophila* Infection

**Figure 1:**
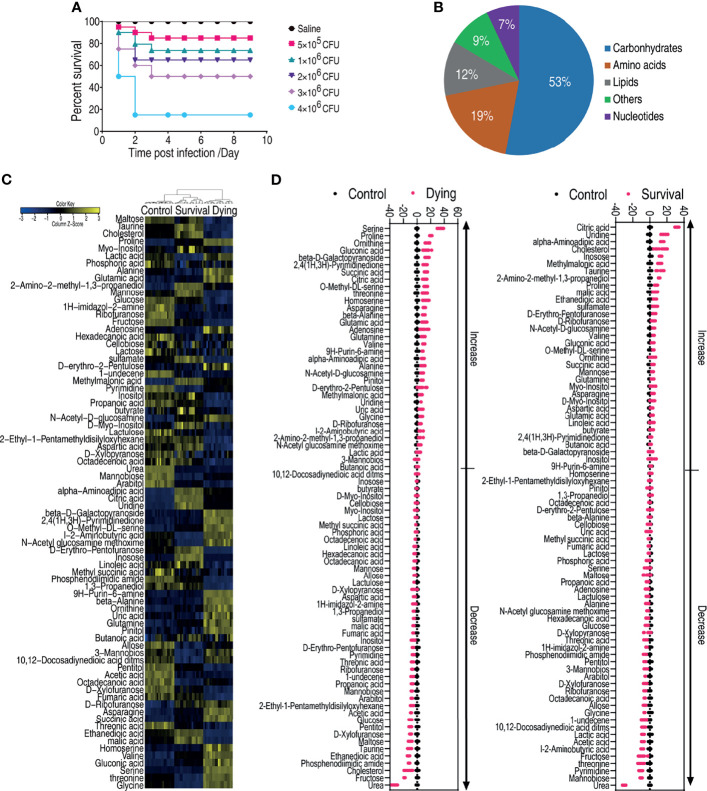
Dying and survival crucian carp have different metabolomic profiles. **(A)** Determination of LD_50_ of crucian carp to *A. hydrophila* infection. **(B)** Functional categories of the differential metabolites. **(C)** Heat map of unsupervised hierarchical clustering of differential metabolites (row). Yellow and blue indicate the increase and decrease of the metabolites scaled to mean and standard deviation of row metabolite level, respectively (see color scale). **(D)** Z scores (standard deviation from average) of the dying group (left panel) or survival group vs. control group (right panel) which correspond to the data shown in **(C)**. Each point represents one technical replicate.

Crucian carp, *Carassius carassius*, were infected with serial doses of *A. hydrophila* (5 × 10^5^, 1 × 10^6^, 2 × 10^6^, 3 × 10^6^, 4 × 10^6^ CFU/fish) by intraperitoneal injection. Fish began to die as early as 24 h postinfection and did not die after 72 h postinfection (**Figure 1A**). The mortality was monitored further for a total of 9 days. Moreover, the dose (3 × 10^6^ CFU) causing 50% of death was selected as LD_50_, which was used for following analysis.

To understand the metabolic signature that determines the consequences of *A. hydrophila* infection, *C. carassius* was challenged with *A. hydrophila* at LD_50_. Ten fish were collected for the dying group, survival group, and control group, respectively. The liver—which not only is considered the core organ for the body to maintain physiological functions but also serves as a major organ in teleost (52)—of each fish was individually removed for gas chromatography–mass spectrometry analysis (GC-MS). Therefore, a total of 60 data points were generated. The internal standard, ribitol, as a control to monitor GC-MS performance and known solvent peaks were removed. The same compounds were merged. The Pearson correlation coefficients between two technical replicates were from 0.9991 to 0.9999 (**Supplementary Figure 1A**), indicating that the technical replicates had good reproducibility. A total of 85 metabolites were identified (**Supplementary Figure 1B**), which fell to five functional categories including carbohydrates (52.9%), amino acids (18.8%), lipids (11.8%), nucleotides (7.1%), and others (9.4%) according to Gene Ontology (GO) (**Figure 1B**). The metabolites were summarized and shown as a heatmap (**Figure 1C**). Interestingly, the three groups were separately clustered, suggesting that the dying fish and survival fish mounted differential response, which were also different from the saline control group (**Figure 1C**). In addition, 78 metabolites of differential abundance were identified. This difference was displayed as a Z score plot in **Figure 1D**, which spanned from -40 to 60 in the dying group including 34 increased metabolites and 44 decreased metabolites and from -20 to 40 in the surviving group including 36 increased metabolites and 42 decreased metabolites. These data together suggest that the metabolome is associated with the state of fish during bacterial infection, highlighting the differential metabolic response in the dying and survival fish.

### Pathway Enrichment Analysis

**Figure 2:**
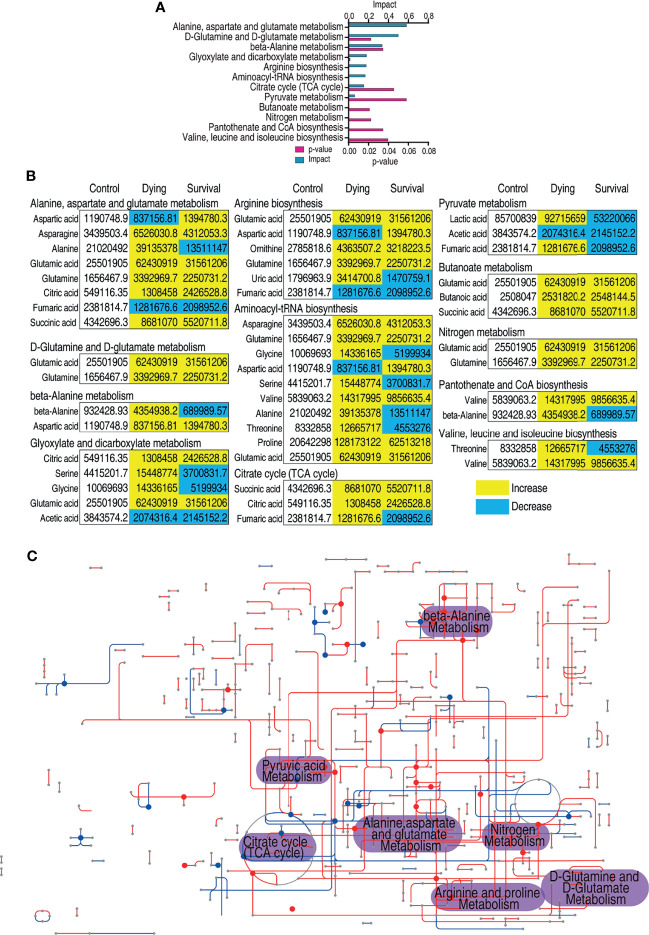
Pathway enrichment analysis of differential metabolites. **(A)** Pathway enrichment analysis of differential metabolites. Significantly enriched pathways were selected to plot (p value <0.05), and their impact score was indicated. **(B)** The relative abundance of metabolites of each pathway listed in **(A)**. Metabolites highlighted with yellow and blue indicate the increased and decreased abundance, respectively. **(C)** Integrated metabolic network of differential metabolites of dying group against survival group by iPath3.0. The red and blue lines represent the increased and decreased metabolites, respectively.

Metabolic pathway analysis provides the route of chemical change offering a comprehensive view to identify the changes during the biological process (53). Thus, the metabolites of differential abundance were searched against MetaboAnalyst 5.0 (54). A total of 12 metabolic pathways were enriched, and the top eight pathways include alanine, aspartate, and glutamate metabolism, glutamine and glutamate metabolism, β-alanine metabolism, glyoxylate and dicarboxylate metabolism, arginine biosynthesis, aminoacyl-tRNA biosynthesis, citrate cycle, and pyruvate metabolism (**Figure 2A**). The metabolites of each pathway are listed in **Figure 2B**. For many of the pathways, the metabolites were accumulated during infection despite that several of the amino metabolites were decreased (**Figure 2B**). However, it is also interesting to notice that most of the metabolites of the dying group increased more than those in the survival group (**Figure 2B**). In addition, iPath provides a global view of the metabolic network by integrating different pathways (55). A significant altered part lies on the central carbon metabolism (**Figure 2C**). Correspondingly, the abundance of the metabolites in the citric cycle was decreased in surviving fish. Moreover, the intermediate metabolite of the TCA cycle accumulated higher in the dying group than those in the survival group except citric acid (**Figure 2B**). The pathway enrichment analysis demonstrates that the dying fish is associated with dysregulated central carbon metabolism.

### Identification of Metabolic Biomarkers Resulting in Dying and Survival

**Figure 3:**
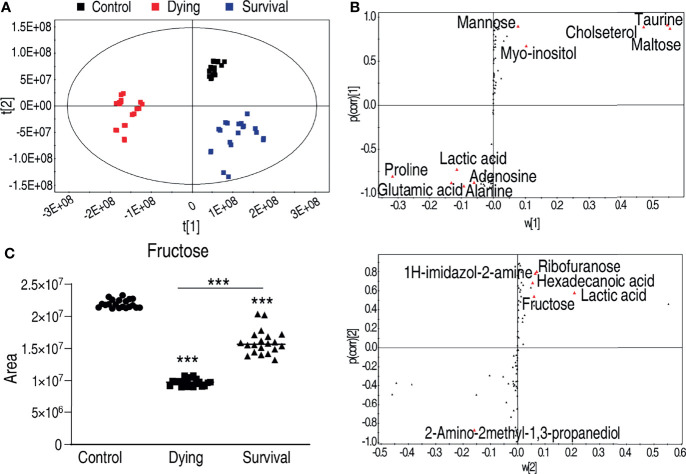
Identification of crucial metabolites that differs survival group from dying group. **(A)** The PCA analysis of the metabolomic data. **(B)** S-plot, generated by PLS-DA, to identify differential metabolites of intragroup as from t[1] and t[2] in **(A)**. The upper panel corresponds to t[1], and the lower panel corresponds to t[2]. Each triangle represents individual metabolite, where potential biomarkers are highlighted with red, which is greater or equal to 0.05 and 0.5 for the absolute value of covariance p and correlation p (corr), respectively. **(C)** The abundance of fructose is the control, dying, and survival groups, respectively. ***p < 0.001.

To identify the crucial metabolite(s) that separate the three groups, partial least square discriminant analysis (PLS-DA) was adopted. The three groups were clearly separated from each other (**Figure 3A**), where R2Y = 0.983 > 0.5, indicating good fitting of the model. Discriminating variables were shown as S-plot for both dying vs. control and survival vs. control, where the cutoff values were set as greater or equal to 0.05 for the absolution value of covariance p, and greater or equal to 0.5 for correlation p(corr) (**Figure 3B**). Crucial biomarkers were identified through component p (1) and are represented with red triangles (**Figure 3B**). Thus, a total of 16 crucial biomarkers were identified and displayed as scatter plots (**Supplementary Figure 2**). Fructose was one of the most outstanding metabolites (**Figure 3C**). Interestingly, the abundance of fructose was decreased in both the dying and surviving groups, but the abundance in the dying group was lower than in the surviving group (**Figure 3C**), implying that fructose might be a survival factor for *A. hydrophila* infection.

### Fructose Enhances *C. carassius* Immune Response and Survival Against *A. hydrophila* Infection

**Figure 4:**
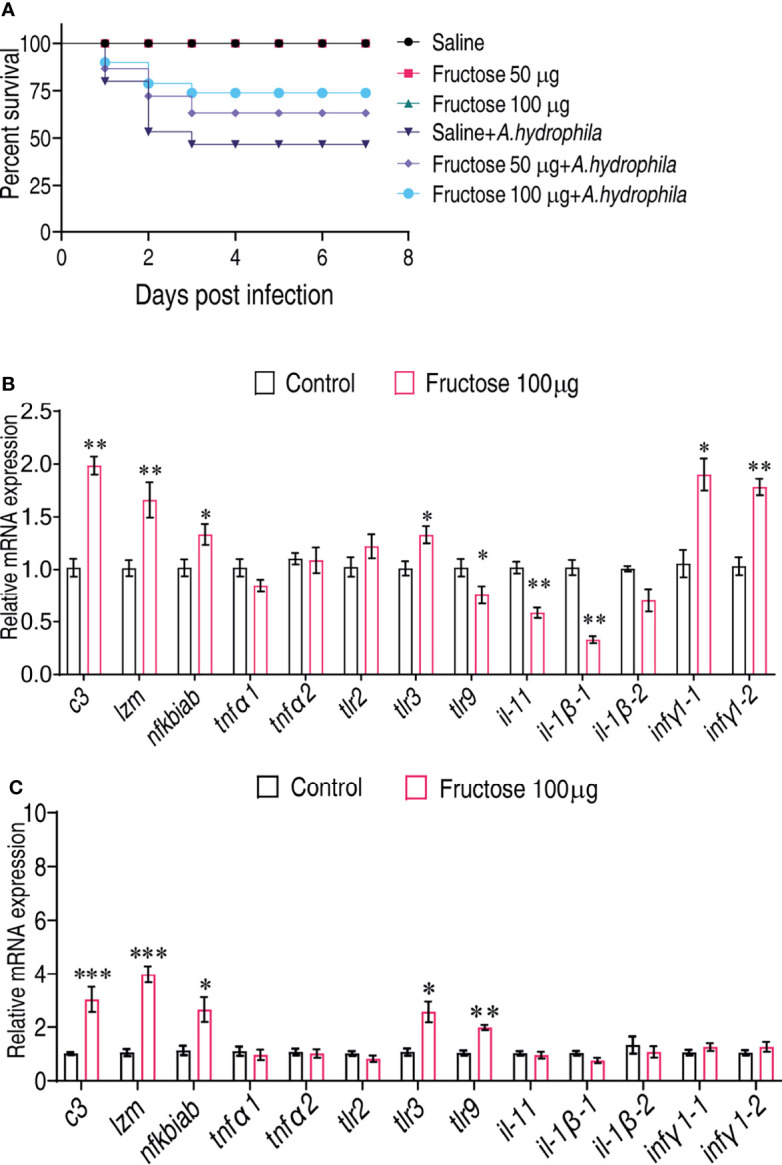
Fructose promote crucian carp survival by modulating immune response. **(A)** Percent survival of crucian carp challenged with *A*. *hydrophila* in the presence or absence of the indicated dose of fructose. **(B)** qRT-PCR for cytokine expression of crucian carp treated with saline or fructose (100 μg) for 3 days. **(C)** qRT-PCR for cytokine genes of crucian carp treated with saline or fructose (100 μg) for 3 days followed by infection with a non-lethal dose of *A*. *hydrophila.* Values are means ± SEM from six biological replicates and significance was analyzed by the Mann–Whitney U-test. *p < 0.05, **p < 0.01, ***p < 0.001.

To examine whether fructose can be a survival-promoting factor, *C. carassius* were injected daily with two concentrations of fructose (50 or 100 μg/fish), which was obtained by scaling up the relative amount to the internal control, ribitol, for 3 days followed by bacterial challenge with *A. hydrophila*. Under these two concentrations, fructose alone had no effect on the survival of *C. carassius* (**Figure 4A**). However, upon bacterial challenge, fructose increased the *C. carassius* survival rate up to 27.2% in a fructose dose-dependent manner (**Figure 4A**), where injection of saline served as control. The relative percent survival rates of the two groups were 37.5% and 56.25% for the groups being treated with 50 and 100 μg/fish, respectively (**Table 1**). The increased fish survival motivated us to investigate whether this potential was driven by immunity. Being treated with 100 µg fructose the same way as mentioned above, the livers were used for immune gene expression analysis. A total of thirteen genes, *tnfα1*, *tnfα2*, *ilb1*, *ilb2*, *lyz*, *ifnγ1-1*, *infγ1-2*, *nfkbiab*, *il11*, *c3*, *tlr9*, *tlr2*, and *tlr3* were included. The expressions of *ifnγ1-1*, *infγ1-2*, *nfkbiab*, *tlr3*, *lyz*, and *c3* were all increased, but others were slightly decreased (**Figure 4B**). In addition, to confirm if the role of fructose on protecting fish against *A. hydrophila* infection correlates with immune response, the fish was challenged with a non-lethal dose of bacteria after being pretreated with fructose as in **Figure 4B**. Consistently, *lyz* and *c3* were the two of the most impacted genes (**Figure 4C**) that increased 4.13- and 2.99-fold, respectively. Thus, these data suggest that fructose influence gene expression that can enhance *C. carassius* to fight against *A. hydrophila* infection.

**Table 1:** Immune protection of fructose to *A. hydrophila* infection in crucian carp.

Treatment	Bacterium	Total fish	Survived^a^	ADR (%)^a^	RPS (%)^a^
Saline	*A. hydrophila*	30	14	53.33	-
Fructose (50µg)	*A. hydrophila*	30	20	33.33	37.5^b^
Fructose (100µg)	*A. hydrophila*	30	23	23.33	56.25^b^

^a^Average of two biological repeats. All the treatment was same volume (5µL). Survived, the number of fish survived in the experiment. ADR, accumulating death rates; RPS, relative percent survival. RPS was calculated as RPS = 1 - (% mortality of Fructose treated group / % mortality of control group) × 100.

^b^P < 0.01.

### Fructose Promotes *C. carassius* to Clear *A. hydrophila*

**Figure 5:**
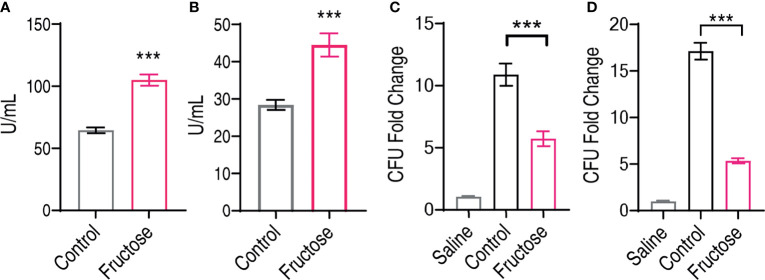
Elevated lysozyme and complement activity in serum and liver can inhibit infected *Aeromonas hydrophila*. **(A)** Lysozyme activity of crucian carp serum being treated with saline or fructose (100 μg). **(B)** Complement activity of crucian carp serum being treated with saline or fructose (100 μg). **(C)** Bactericidal effect of serum treated with saline or fructose (100 μg). **(D)** Bactericidal effect of liver homogenates treated with saline or fructose (100 μg). All the data were plotted as histogram, and each dot represents one biological replicate. Scatter plot represents mean ± SEM of 10 biological replicates, significance was analyzed by the non-parametric Mann–Whitney U-test, ***p < 0.001.

Fructose promotes the expression of innate humoral resonance including *lyz* and *c3*, and these two molecules are known for their bacterial lytic activity (56, 57). We postulate that fructose promotes the serum level of lysozyme and complement component 3 that help the elimination of bacteria, which can also be used to confirm our qRT-PCR results. Thus, we measured the serum level of *C. carassius* after being treated with 100 µg fructose. The lysozyme activity was increased from 64.53 ± 2.31 to 105.48 ± 4.51 U/ml, and the complement activity was increased from 28.55 ± 1.36 to 45.53 ± 3.11 U/ml (**Figures 5A, B**). Moreover, these data corroborate our qRT-PCR data that fructose increased the expression of lysozyme and C3 at both transcriptional level and protein levels. Moreover, we also analyzed whether the increased lysozyme and C3 contribute to the cell killing. Bacteria were incubated with saline, serum, or serum isolated from fructose-treated fish (fructose-serum) for cell killing. As compared to the saline control, the bacteria grew dramatically in the control, whose CFU increased 10.36-fold, whereas the growth in the fructose serum was slowed for 1.9-fold as compared to serum alone (**Figure 5C**). Meanwhile, the liver extracts were also applied. Surprisingly, the growth of the liver extracts from fish pretreated with fructose (fructose-liver extracts) was 5.35-fold slower than the liver extracts alone (**Figure 5D**). These data together suggest that fructose increased the humoral lytic ability toward *A. hydrophila.*

### Fructose Enhances Bacterial Clearance *In Vivo*

**Figure 6:**
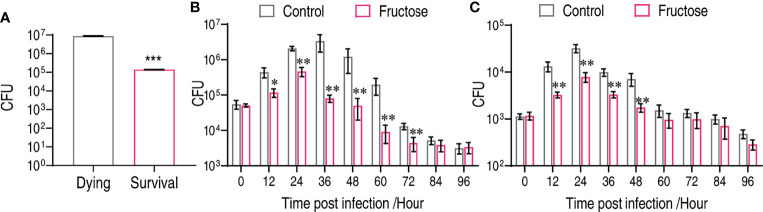
Fructose reduce bacterial load *in vivo*. **(A)** CFU counts in dying (n = 15) and survival (n = 15) liver of crucian carp. **(B)** Time-dependent CFU count in the liver of crucian carp that were treated with fructose (100 μg) for 3 days and infect with lethality dose of bacteria. Scatter plot represents mean ± SEM of 6 biological replicates, significance was analyzed by Mann–Whitney U-test, *p < 0.05, **p < 0.01, ***p < 0.001. **(C)** Dynamic change of crucian carp liver bacterial number after being treated with or without fructose (100 μg) for 3 days and infected with a non-lethal dose of bacteria. Scatter plot represents mean ± SEM of 6 biological replicates; significance was analyzed by the Mann–Whitney U-test, **p < 0.01, ***p < 0.001.

To support the fructose-mediated bacterial clearance *in vivo*, we firstly established that the number of bacteria in the survival fish was around 63.31-fold lower than that in the dying fish (**Figure 6A**). The number of viable bacteria was a critical factor for fish survival. *C. carassius* were treated with either LD50 or a non-lethal dose of bacteria so that the role of fructose in reducing bacterial load can be revealed. Under an LD50 dose challenge, the control group had an increased number of bacteria in 36 h after intraperitoneal infection, which increased 61.44-fold after 36 h postinfection (**Figure 6B**). However, the number in fructose-treated fish was increased less than 9.17-fold at the first 24 h but decreased afterward (**Figure 6B**). Similarly, upon non-lethal bacterial challenge, fructose was also more potent on decreasing bacteria number than by the immune system itself (**Figure 6C**). Thus, these *in vitro* data demonstrate that fructose enable bacteria clearance that can be further explored in aquaculture.

## Discussion

Combating bacterial infection is required for the sustainable development of aquaculture that is of increasing importance to provide the protein sources to end hunger (58, 59). In addition to the currently developed strategies, boosting the host’s immunity by metabolites is highly appreciated due to their eco-friendly, inexpensive, and nutritional characteristics (20). Moreover, these characteristics are crucial in aquaculture since contamination to the environment may cause severe consequences to both society and human health, e.g., the residual antibiotics in the environment cause the emergence of antibiotic-resistant bacteria and the spread of antibiotic genes (60, 61). Therefore, the search for the appropriate metabolites is urgently required.

The metabolome is the sum of all the biological processes that take place at a certain time. Therefore, profiling the metabolites that are altered at different biological conditions has implications for the adapted metabolism. Likely, the dying fish and surviving fish upon bacterial infection represent different biological phenotypes, which can also be identified at the metabolomic level, and metabolites that distinguish the two phenotypes can be drawn, enabling the identification of metabolites in promoting fish survival. Therefore, our study established the metabolomic change of *C. carassius* during infection, and more importantly, the metabolomes of dying and survival fish can be separated. Our study suggested that metabolomic analysis not only can be diagnostic to bacterial infection but also sheds light on altering metabolic pathways that can be harnessed to enhance host survival. In this study, for example, we proposed that the hyperactive pyruvate metabolism was correlated with fish death. The metabolites, lactic acid, and fumaric acid were accumulated in dying fish. Since both lactic acid and fumaric acid are anti-inflammatory (62, 63), it is highly possible that the inflammation was not sufficient at the beginning of infection, so that the fish fails to clear the bacteria that ultimately overwhelm *C. carassius*’s immunity.

Fructose is known as a pro-inflammation metabolite in mammals (64). The excessive intake of fructose is harmful for health, especially for organisms with chronic diseases (65–67). High fructose (10.5 g/kg) (in our study, fructose was administrated with 50 mg/kg) significantly increases uric acid and proinflammatory cytokines in serum, such as IL-6, TNF-α, and MIP-2, and decreases IL-10, an anti-inflammatory cytokine (68). Rats administrated with fructose by drinking water at 7.8 mg/kg for 15 weeks increased iNOS and *tnfα* mRNA but decreased *il-10* and *il-6* mRNA (69). However, these studies were taken on non-alcoholic steatohepatitis and non-alcoholic fatty liver diseases. Another study shows that fructose promotes the expression of cytokines *via* its metabolite, FBP (fructose-1,6-bisphosphate/fructose-2,6-bisphosphate) (19, 64, 70). Although fructose represses the cytokine expression in our study, we indeed found that fructose enhances the expression of innate humoral molecules including lysozyme and c3 at both transcriptional and protein levels. This may be due to the pretreatment of fish with fructose. Unlike the previous study, they demonstrate that the coadministration of fructose and lipopolysaccharide enhanced the expression of cytokines (64). The treatment with fructose alone may not be able to trigger cytokine expression unless pathogen-associated molecules were present. Since the regulatory mechanisms of lysozyme and c3 expression were not fully understood even in mammals, we failed to explore how fructose regulates lysozyme and C3, which requires further investigation. The identification of fructose as a survival-promoting factor was interesting. The previous identification of maltose and glucose as survival-promoting factors in grass carp and tilapia, respectively, suggests that carbohydrates are an important ingredient in fish diet, but the dosage is critical.

One limitation of this study is that we only focused on the gene expression of certain genes, which may omit other important molecules, especially lysozyme and complement component 3 which might partially contribute to survival; moreover, antimicrobial peptides, c-reactive protein, protease inhibitors, pentraxins, and transferrin (71), which are also known to participate in the defense of bacterial infection, should be further investigated. Nonetheless, whether fructose can potentiate the antibacterial property of IgT-dependent bacterial clearance was of interest for further investigation, even though to our knowledge there is no report regarding the association of fructose and adaptive immune response at higher vertebrates.

**Figure 7:**
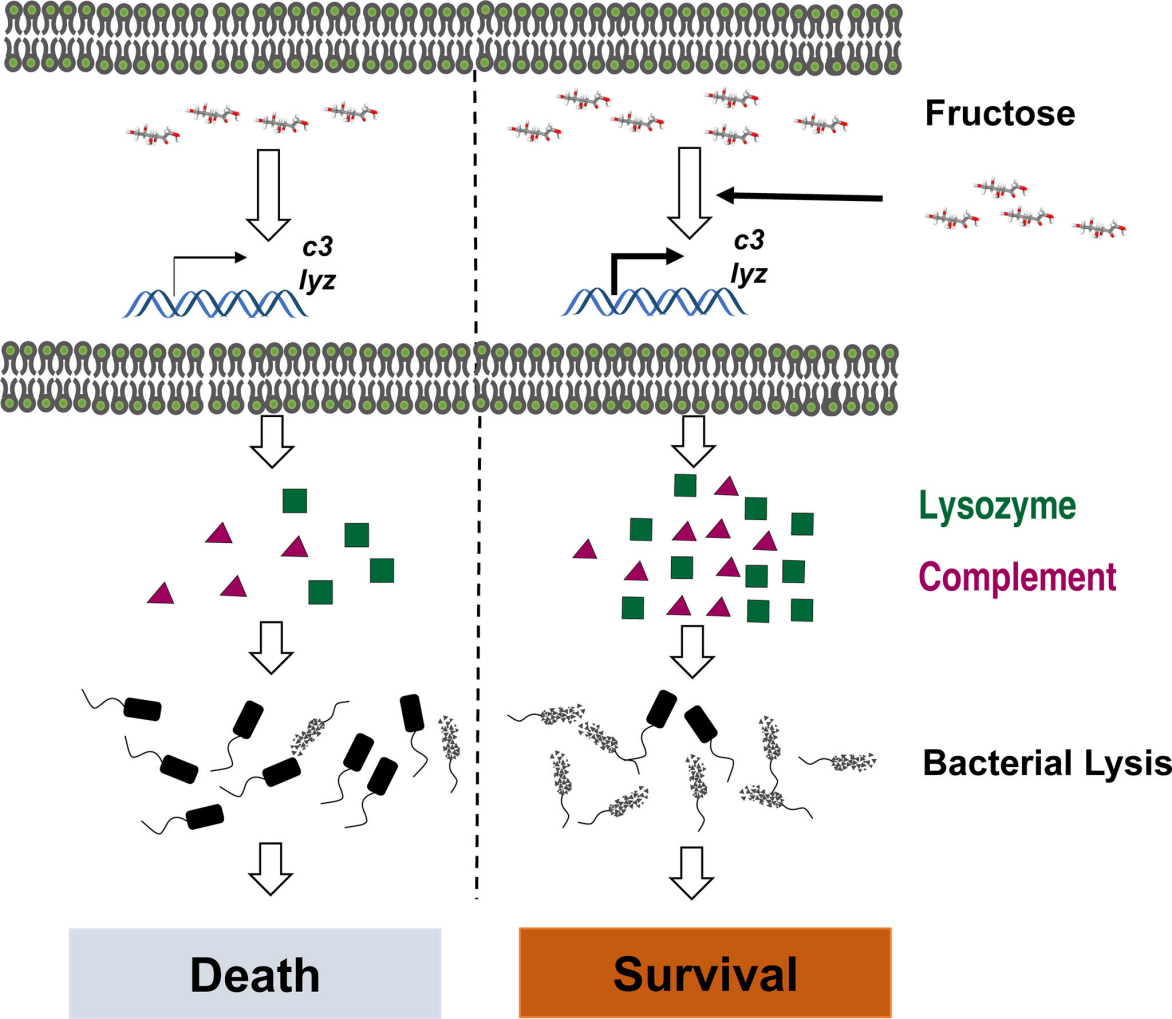
Graph summary of fructose promoting crucian carp survival against *A. hydrophila* infection.

In summary, we established the metabolic signature of *C. carassius* upon *A. hydrophila* infection and identify fructose as a metabolite in promoting *C. carassius* survival. We also report that fructose mainly enhances the expression of lysozyme and C3, which enable the bacteria to be reduced more efficiently (**Figure 7**), thus highlighting a metabolism-based approach to combat bacterial infection in aquaculture.

## Data Availability Statement

The original contributions presented in the study are included in the article/**Supplementary Material**. Further inquiries can be directed to the corresponding author.

## Ethics Statement

Animal studies involved in this manuscript adhere to the recommendations in the Guide for the Care and Use of Laboratory Animals of the National Institutes of Health and maintained according to the standard protocols. All the experiments were reviewed and approved by the Institutional Animal Care and Use Committee of Sun Yat-sen University (Approval No. SYSU-IACUC-2020-B126716).

## Author Contributions

YC and TK conducted the experiments. YC, TK, and LP performed the data analysis. YC, TK, LP, and HM interpreted the data. BP wrote the manuscript. BP conceptualized and designed the project. All the authors reviewed the manuscript and acknowledged the contributions. All authors contributed to the article and approved the submitted version.

## Funding

This work was sponsored by grants from National Natural Science Foundation of China (Nos. 31872602, 32061133007, 31822058), project supported by Innovation Group Project of Southern Marine Science and Engineering Guangdong Laboratory (Zhuhai) (No. 311020006), The Youth Talent Support Program of Guangdong Province (No. 2017GC010617) to BP, and fundings from the Research Council of Norway Grant No. 320692 (to HM).

## Conflict of Interest

The authors declare that the research was conducted in the absence of any commercial or financial relationships that could be construed as a potential conflict of interest.

## Publisher’s Note

All claims expressed in this article are solely those of the authors and do not necessarily represent those of their affiliated organizations, or those of the publisher, the editors and the reviewers. Any product that may be evaluated in this article, or claim that may be made by its manufacturer, is not guaranteed or endorsed by the publisher.
