# Supplementary material for: Fructose Promotes Crucian Carp Survival Against Aeromonas hydrophila Infection
Source: Front Immunol. 2022 Mar 21;13:865560. doi: 10.3389/fimmu.2022.865560 (PMC8979172; doi:10.3389/fimmu.2022.865560)
Supplement: Supplementary file 1 [file DataSheet_1.docx]

Supplementary Figure


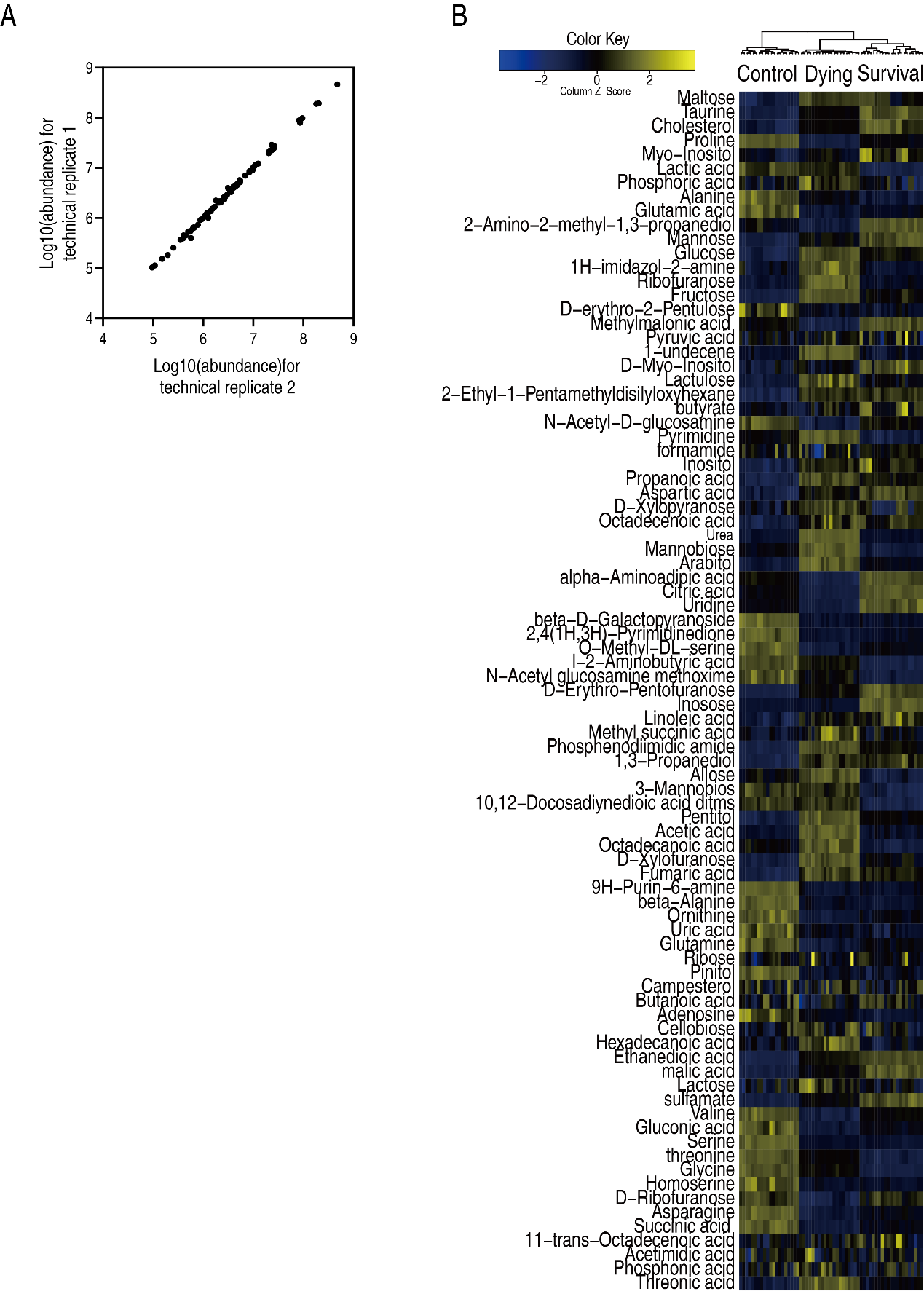


Figure 1 Golbal metabolites heatmap and technincal correlations

A：Reproducibility of metabolic profiling platform. This plot shows two technical replicates with the weakest Pearson correlation coefficient.

B：Heat map of unsupervised hierarchical clustering of global metabolites (row). Yellow and blue indicate the increase and decrease of the metabolites scaled to mean and standard deviation.


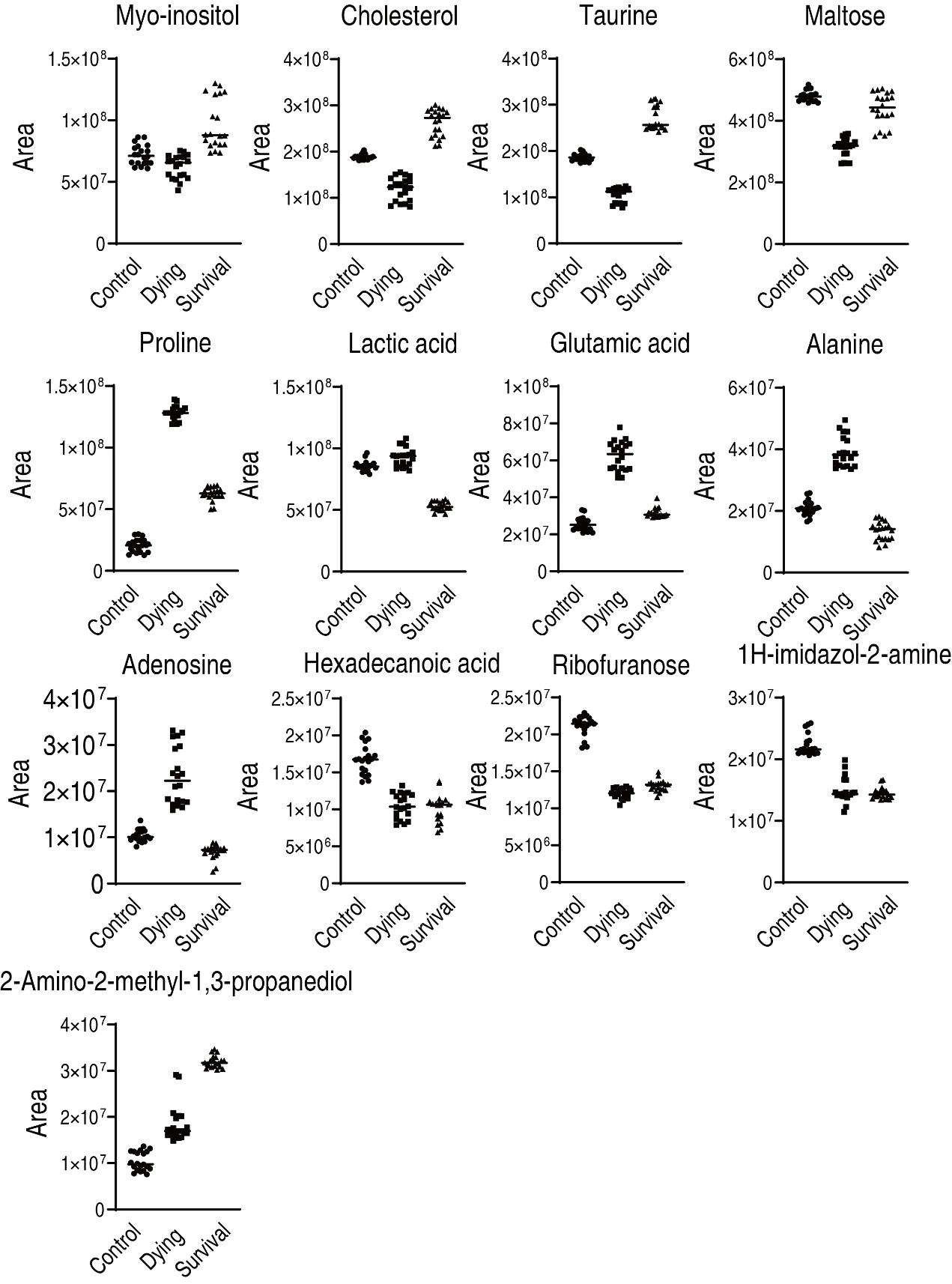


**Figure 2 Total crucial biomarkers screened by PLS-DA which can distinguish control, dying-group and survival-group**
